# Psychometric validation of the 15-item Questionnaire about the Process of Recovery in Spain (QPR-15-SP)

**DOI:** 10.3389/fpsyg.2023.1178341

**Published:** 2023-07-05

**Authors:** Jessica Marian Goodman-Casanova, Daniel Cuesta-Lozano, Catalina Alupoaei, Eva María Grasa Bello, Jesús Herrera-Imbroda, Fermin Mayoral-Cleries, Jose Guzman-Parra

**Affiliations:** ^1^Unidad de Gestión Clínica de Salud Mental, Hospital Regional Universitario de Málaga, Instituto de Investigación Biomédica de Málaga (IBIMA), Málaga, Spain; ^2^Departamento de Enfermería y Fisioterapia, Universidad de Alcalá, Madrid, Spain; ^3^Mental Health, Institut d’Investigació Biomèdica Sant Pau (IIB SANT PAU), Barcelona, Spain; ^4^Centro de Investigación Biomédica en Red de Salud Mental (CIBESAM), Madrid, Spain; ^5^Grupo de Neuropsicofarmacología, Hospital Regional Universitario de Málaga - Instituto de Investigación Biomédica de Málaga y Plataforma en Nanomedicina–IBIMA Plataforma Bionand, Málaga, Spain; ^6^Departamento Farmacología y Pediatría, Facultad de Medicina, Universidad de Málaga, Málaga, Spain

**Keywords:** mental health recovery (MeSH), outcome and process assessment (health care) (MeSH), psychometrics (MeSH), psychotic disorders (MeSH), surveys and questionnaires (MeSH)

## Abstract

**Introduction:**

Reliable and valid instruments are needed to measure the impact of mental health services and programs on the journeys of recovery of service users. The aim of this study was to explore the psychometric properties of the cross-culturally adapted 15-item Questionnaire about the Process of Recovery in Spain (QPR-15-SP).

**Methods:**

One hundred and ten participants from three locations in Spain (Málaga, Barcelona and Madrid), who were users of primary and specialized mental health services, were interviewed from October 2021 to June 2022.

**Results:**

The internal consistency obtained was excellent: *ω*  =.93 and *α* =.92. Temporal reliability using intraclass correlation coefficients was moderate (ICC=.684, *p* <.000). Regarding convergent validity, the QPR-15-SP had a moderate correlation with the Clinical Outcomes in Routine Evaluation-Outcome Measure (CORE-OM) (*ρ* =-.500, *p* <.000), a Visual Numeric Recovery Scale (VNRS) (*ρ* =.591, *p* <.000), and the Stages of Recovery Instrument (STORI) (*r* =.566, *p* <.000). Correlations between advanced stages of recovery and higher QPR-15-SP scores were found (Moratorium: *ρ*  =-.579, *p* <.000; Awareness: *ρ* =-.130, *p* =.189; Preparation: *r* =-.043, P=.665; Rebuilding: *r* =.460, *p* <.000; Growth: *ρ*  =.697, *p* <.000). In terms of divergent validity, the QPR-15-SP had low correlation with the DUKE-UNC Functional Social Support Scale (*ρ* =.273, *p* <.005). The confirmatory factor analysis of the 1-factor structure obtained reasonable goodness of fit indexes.

**Discussion:**

The QPR-15-SP has acceptable psychometric properties, providing support for measuring recovery in Spain and allowing international comparison research.

## 1. Introduction

The current concept of recovery in mental health is the result of historical intellectual, social, and political movements that questioned the mental health-mental illness dichotomy, the etiology of mental health problems, the role of and the relationship between people and mental health professionals and the organization of mental health services (Desviat, 2020). As a result of this evolution, the understanding of recovery goes beyond clinical improvement as a passive result of the reduction or absence inernalof psychopathological symptoms (biomedical model) and the mere functional adaptation of the person to society (psychiatric rehabilitation model) to a process that involves engaging actively in meaningful experiences even in the presence of mental health problems (recovery model) (Braslow, 2013).

The recovery-oriented model has become a cornerstone in mental health over the last decades (Frost et al., 2017), shifting from a service-based symptom remission approach to a holistic community-based person-centered framework (Jørgensen et al., 2021). Mental health policies are moving towards culturally tailored programs and services which promote user involvement and measure user-defined recovery-oriented outcomes. Intersectoral care plays an important role in enhancing an active ongoing journey of recovery within local communities and boosting social support (Bjørlykhaug et al., 2021).

Despite the existence of commonly accepted definitions and descriptions of processes, stages and domains (Leendertse et al., 2021), recovery continues being a complex and multidimensional notion with cultural heterogeneity and great variability in its conceptualization and evaluation (Penas et al., 2019).

Understanding the specific impact of culture in recovery is essential for recovery-oriented services and programs (Slade et al., 2014) because it allows clinicians and researchers to identify the areas of care that need focus and attention. The role of social support has been described as a key coping resource (Turner and Brown, 2010), studies have explored how social support may promote recovery (Corrigan and Phelan, 2004) and a recent review has studied the linkage between recovery and social support (Bjørlykhaug et al., 2021). Studies have shown that Spain has an interdependent culture with a social and relational character (Saavedra et al., 2022). In fact, studies in Spain show that recovery is mostly related with the clinical aspects of recovery and socializing, and least related with wellbeing and resistance (Saavedra et al., 2022).

A framework for understanding recovery is the CHIME framework which stands for the recovery processes of Connectedness, Hope and optimism about the future, Identity, Meaning, and Empowerment (Leamy et al., 2011). This framework has proven to be internationally valid to understand recovery (Slade et al., 2012; Bird et al., 2014) This framework has allowed the evaluation of the extent to which instruments measure recovery internationally (Shanks et al., 2013), and nationally (Penas et al., 2020).

The service-user movement has moved away from professionally developed and administered measures, to self-reports of user-defined outcomes as a result of the alliance between experts by profession and experts by experience (Slade and Longden, 2015). The Human Rights, Antipsychiatry and Psychiatry Survivors movements resulted in the consideration of people as active participants in a process of self-determination and resignification of life beyond mental illness and as experts in their mental health experiences, defining mental health recovery in their own terms (Braslow, 2013).

Out of all the recovery instruments, the second best instrument with psychometric properties, and the only recovery measure to fully correlate with the CHIME framework (Shanks et al., 2013), reported as user-friendly (Law et al., 2012), and developed in collaboration with service users is the Questionnaire about the Process of Recovery (QPR) (Neil et al., 2009).

The original QPR is a 22-item measure with two subscales: “Intrapersonal” concerning tasks that the person is responsible for to rebuild a life, and “Interpersonal” in terms of the ability of reflection of the person of their value in the external world and on the influence of external processes and interpersonal relationships in recovery (Neil et al., 2009). A shortened version of 15 items of the QPR (QPR-15) showed better psychometric properties than the original version (Law et al., 2014). The original English version has been validated in Chinese (Chien and Chan, 2013), Swedish (Argentzell et al., 2017), and Japanese (Kanehara et al., 2017, 2020), and the 15-item version has been adapted in Spanish (QPR-15-SP) (Goodman-Casanova et al., 2022).

The QPR-15-SP followed a rigorous process of cross-cultural adaptation in Spain, has conceptual, linguistic, cultural, and metric equivalence with the original QPR-15; and stands out for its comprehensibility, clarity and briefness in length with a completion time of 3 minutes (SD 1.95) (Goodman-Casanova et al., 2022).

Interest in the advancement of recovery measures in Spain is ongoing, as demonstrated by the growing body of evidence in this research area. The only validated measure in Spain when this research was initiated in 2019 was the STORI (Lemos-Giráldez et al., 2015). Recent studies have validated two other recovery measures: the Recovery Enhancing Environment (REE) which measures the recovery orientation of mental health services in general, and the subscale of recovery markers, the moment of personal recovery in particular (Uriarte et al., 2020); and the Recovery Assessment Scale (RAS-24) which measures personal recovery (Saavedra et al., 2021). Both have 24 items, are measured on a 5 Likert-type scale, and higher scores are indicative of greater recovery.

Reliable and valid instruments are needed to measure the impact of mental health services and programs on the journeys of recovery of service users (Keet et al., 2019). The aim of this study was to explore the psychometric properties of the cross-culturally adapted 15-item Questionnaire about the Process of Recovery in Spain. The specific objectives of this study were to assess the reliability of the questionnaire in terms of internal consistency and temporal reliability and to analyze the validity of the questionnaire in terms of criterion and construct validity.

## 2. Materials and methods

### 2.1. Design

The psychometric validation of the QPR-15-SP followed the methodological recommendations described by Ramada-Rodilla and Serra-Pujadas (2013) and the standards of the International Society for Quality of Life Research (ISOQOL) for patient-reported outcome (PRO) measures (Reeve et al., 2013).

### 2.2. Participants

One hundred and ten Spanish service users, with a history of psychosis from primary and specialized mental health services, from three locations in Spain were interviewed for this multicenter study. Participants were referred from four hospitals in Andalucía, Madrid and Barcelona; from a Public Foundation for the Social Integration of People with Mental Illness, and an Association of Relatives and People with Mental Illness.

Primary mental health services covered Community Mental Health Services (*n* = 16) and specialized services included Recovery-oriented Mental Health Services (Day Hospital (*n* = 13), Rehabilitation Unit (*n* = 11), Homeless and Outreach Mental Health Services (*n* = 10), Outpatient Services (*n* = 7), Day Center (*n* = 4), and Inpatient Therapeutic Community (*n* = 2)), a Public Foundation for the Social Integration of People with Mental Illness (*n* = 39) and an Association of Relatives and People with Mental Illness (*n* = 8).

#### 2.2.1. Eligibility criteria

The inclusion criteria for the study were: 18 years or older; a diagnosis of block F20-F29: Schizophrenia, schizotypal and delusional disorders or F30–F39 Mood [affective] disorders with a psychotic history according to the International Classification of Diseases 10th revision (ICD-10) (which is the revision in force in the Spanish Health Service); scoring over 50 points on the Global Assessment of Functioning (GAF); and agreeing to participate by giving signed written consent.

The GAF evaluates the overall performance on the current psychological, social and occupational status on a scale of 0 to 100 points were scores under 50 indicate severe problems in functioning (Hall, 1995). The Spanish version was used (Servicio Andaluz de Salud, 2010).

### 2.3. Instruments

As there is no currently available gold standard to measure recovery, the QPR-15-SP scores were correlated with the Stages of Recovery Instrument (STORI), the Clinical Outcomes in Routine Evaluation-Outcome Measure (CORE-OM), and a Visual Numeric Recovery Scale (VNRS) for convergent validity, and the Duke-UNC Functional Social Support Scale (DUKE-UNC) for divergent validity.

The QPR-15 is a self-administered recovery questionnaire with 15 items scored from 0 (strongly disagree) to 4 (strongly agree) with a maximum score of 60, where higher scores are indicative of recovery (Law et al., 2014). The Spanish cross-culturally adapted version, QPR-15-SP, was used (Goodman-Casanova et al., 2022) (Supplementary material).

The Stages of Recovery Instrument (STORI) is a 50-item self-report evaluating different stages of the recovery process (Moratorium, Awareness, Preparation, Rebuilding, and Growth), where the person is situated in that stage in which he/she obtains the highest score (Andresen et al., 2006). The Spanish version used has shown adequate psychometric properties: internal consistency ranged between 0.83 and 0.87, the three-cluster model fitted the data better than the five-cluster model, and the STORI stages were associated with the Recovery Styles Questionnaire (RSQ) scores (Lemos-Giráldez et al., 2015).

The Clinical Outcomes in Routine Evaluation-Outcome Measure (CORE-OM) is a self-administered questionnaire that evaluates domains of psychological distress: subjective well-being, problems/symptoms, general functioning, and risk. Scores range from 0 (mild) to 25 (severe) distress (Evans et al., 2000; Barkham et al., 2006). The Spanish version used has shown adequate psychometric properties: internal consistency ranged between 0.7 and 0.9, the test–retest stabilities for all domains ranged between 0.76 and 0.87, except for the Risk domain which was 0.45, and the CORE-OM domain scores were associated with the Beck Depression Inventory-II (BDI-II) and the Symptom Checklist 90 Revised (SCL-90-R) (Feixas et al., 2016).

Following the methods of other recovery validation studies (Biringer and Tjoflåt, 2018), a Visual Numeric Recovery Scale (VNRS) was used. The VNRS is a one-dimensional scale on recovery defined as “the development of a new meaning and new goals in personal life, beyond the impact of mental illness” measured from 0 (the worst I’ve been in my recovery journey) to 10 (the best I imagine I could be in my recovery journey).

The Duke-UNC Functional Social Support Questionnaire (DUKE-UNC) is a self-report questionnaire of 11 items to measure perceived social support. Each item is scored using a 5 Likert-type scale of 1 (much less than I would like) to 5 (as much as I would like) with a score ranging between 11 and 55 points (Broadhead et al., 1988). In the Spanish validation, a cut point was chosen in the 15th percentile, which corresponds to a score lower than 32 indicating a low perceived social support and a score equal to or greater than 32 indicating a normal support (Bellón Saameño et al., 1996).

### 2.4. Procedure

The study was presented to professionals of the participating institutions via face-to-face meetings. Participants who met the eligibility criteria were referred by their care coordinators. Researchers invited participants to take part in the study by explaining the Participant Information Sheet which included: the objective of the study, the voluntary nature of participation, and compliance with the legislation on data protection. Participants then gave written consent by signing a Consent Form. Participants were asked to complete all instruments at baseline, and the QPR-15-SP again at 2 weeks. Interviews were carried out between October 2021 and June 2022 individually by trained researchers, and data was subsequently digitized.

### 2.5. Data analysis

Descriptive statistics considered for presentation were the characteristics of the participants and the scores of the instruments. Statistics for continuous measures such as the mean, and standard deviation were presented in summary tables. Categorical variables were summarized using counts and percentages. To study reliability, internal consistency was calculated according to McDonald’s Omega and Cronbach’s alpha. Intraclass correlation coefficients (ICC) were used to explore temporal reliability. For the validity, Pearson correlations were used for normally distributed variables and Rho Spearman correlation for the rest. A confirmatory factor analysis (CFA) was carried out to assess the 1-factor solution obtained in the original validation study (Law et al., 2014), using the weighted least-squares mean variance (WLSMV) estimator with the package Lavaan (Rosseel, 2012). To assess the goodness of fit the following indices were used: *X*^2^ (*p* > 0.05), comparative fit index (CFI > 0.95), Tucker–Lewis Index (TLI > 0.95), root mean square error of approximation (RMSEA < 0.06), and standardized root mean square residual (SRMR < 0.08). R (version 4.2.1, The R Foundation) was used for all statistical analysis (R Core Team, 2021).

## 3. Results

### 3.1. Characteristics of participants

One hundred and four mental health service users who were mostly single men with a mean age of 45.89 (SD 11.63), had secondary school studies, lived with their family of origin and received government financial aid participated. Participants were mostly recruited in Recovery-oriented Mental Health Services, were diagnosed of an F20–29 diagnosis, had an average of 20.17 (SD 12.07) years of experience in mental health, and had had 4.25 (SD 5.867) hospital admissions during their lifetime. Detailed sociodemographic characteristics are presented (Table 1). One hundred and ten participants were recruited, out of which data for six users, who did not complete the full assessment, was excluded from the analysis. All six users decided not to carry on with the assessment when completing the STORI reasoning it was tedious to complete. There is missing data for four participants which could not be located in time for the timeframe established.

**Table 1:** Characteristics of participants.

	Total (*N* = 104)
Age M(SD)	45.89 (11.63)
Sex, *n* (%)	
Man	74 (71.2)
Woman	30 (28.8)
Marital status, *n* (%)	
Single	81 (77.9)
Married/Couple	9 (8.6)
Separated/Divorced	13 (12.5)
Widowed	1 (1)
Level of education, *n* (%)	
Less than elementary	2 (1.9)
Elementary school	22 (21.2)
Secondary school	38 (36.5)
Higher education	29 (27.9)
University education	13 (12.5)
Living arrangements, *n* (%)	
Alone	31 (29.8)
With parents and/or siblings	42 (40.4)
With spouse and/or children	11 (10.6)
Residential home	10 (9.6)
Sharing with others	10 (9,6)
Employment status, *n* (%)	
Employed	3 (2.9)
Unemployed	14 (13.5)
Government financial aid	83 (79.8)
Others	4 (4)
Mental health services, *n* (%)	
Community Mental Health Services	14 (13.5)
Recovery-oriented Mental Health Services	47 (45.1)
Mental Health Social Services	36 (34.6)
Mental Health Service-User Association	7 (6.7)
ICD-10, *n* (%)	
F20–29 Schizophrenia, schizotypal and delusional disorders	88 (84.6)
F30–39 Mood [affective] disorders	16 (15.4)
Years in follow-up by mental health services M(SD)	20.17 (12,074)
Times admitted in hospital, M(SD)	
Lifetime	4.25 (5.867)
Last year	0.19 (0.801)

### 3.2. Descriptive statistics

The total score of the QPR-15-SP was 41.77 (SD 11.143) and scores ranged from 2.31 (item 4) to 3.06 (item 15) (Table 2). Negative values for the skewness indicate that data was slightly skewed left, and kurtosis shows a platykurtic distribution (Table 2).

**Table 2:** Descriptive statistics of the 15 items of the QPR-15-SP.

Item	Range	Mean	Standard deviation	Skewness	Kurtosis
Item 1	0–4	2.69	1.158	−0.787	−0.203
Item 2	0–4	2.81	1.025	−0.818	0.229
Item 3	0–4	2.87	1.089	−1.108	0.842
Item 4	0–4	2.31	1.270	−0.456	−0.840
Item 5	0–4	2.65	1.095	−0.493	−0.577
Item 6	0–4	2.83	1.038	−0.974	0.799
Item 7	0–4	3.03	1.092	−1.197	0.788
Item 8	0–4	2.98	1.106	−1.323	1.231
Item 9	0–4	2.91	1.089	−0.882	0.021
Item 10	0–4	2.9	1.093	−1.080	0.649
Item 11	0–4	2.69	1.107	−0.939	0.377
Item 12	0–4	2.62	1.126	−0.647	−0.375
Item 13	0–4	2.71	0.982	−0.706	0.271
Item 14	0–4	2.71	1.002	−0.690	0.129
Item 15	0–4	3.06	0.964	−1.179	1.191

### 3.3. Reliability

#### 3.3.1. Internal consistency

Internal consistency, using McDonald’s Omega and Cronbach’s alpha was excellent (*ω* = 0.93 and *α* = 0.92).

#### 3.3.2. Temporal reliability

The mean total score for those who completed both assessments was 42.01(SD 11.151) at baseline, and 41.66 (SD 10.118) at 2 weeks. Temporal reliability using intraclass correlation coefficients was moderate (ICC = 0.684, *p* < 0. 000).

### 3.4. Validity

#### 3.4.1. Criterion validity

Sample scores on study measures are provided (Table 3). Regarding convergent validity, the QPR-15-SP had a moderate Pearson correlation with CORE-OM (*ρ* = −0.500, *p* < 0.000) and with VNRS (*ρ* = 0.591, *p* < 0.000), and a moderate Rho de Spearman correlation with STORI (*r* = 0.566, *p* < 0.000). A correlation between advanced stages of the STORI and higher QPR-15-SP scores was found (Moratorium: *ρ* = −0.579, *p* < 0.000; Awareness: *ρ* = −0.130, *p* = 0.189; Preparation: *r* = −0.043, *p* = 0.665; Rebuilding: *r* = 0.460, *p* < 0.000; Growth: *ρ* =0.697, *p* < 0.000). In terms of divergent validity, the QPR-15-SP had low Pearson correlation with the DUKE-UNC (*ρ* = 0.273, *p* < 0.005).

**Table 3:** Sample scores on study measures.

	Range	Mean	Standard deviation
QPR-15-SP	39.60–43.94	41.77	11.143
CORE-OM	58.31–66.90	62.60	22.10
VNRS	6.4–7.31	6.86	2.33
STORI			
Moratorium	15.68–20.45	18.07	12.25
Awareness	25.33–30.61	27.97	13.56
Preparation	27.48–32.49	29.98	12.88
Rebuilding	34.14–37.76	34.14	9.305
Growth	33.25–37.88	35.57	11.91
DUKE-UNC	40.17–44.15	42.16	10.227

#### 3.4.2. Construct validity

The CFA indices approach acceptability for the one factor solution: *X*^2^ (90) = 179.920, *p* < 0.01, CFI = 0.944, TLI = 0.935, RMSEA = 0.098 (90% CI, 0.077–0.119), and SRMR = 0.073.

## 4. Discussion

Given the global emphasis on measuring mental health service effectiveness (World Health Organization, 2013) and using routine recovery outcomes in clinical practice (Healthcare of Health Social Services and Equality, 2009) the QPR-15-SP introduces a valuable and meaningful asset for the assessment of people in their recovery journeys with a reliable and valid version for use in the Spanish language and cultural context.

This study stands out methodologically in psychometrically validating a recovery measure. In comparison with other international and national studies on recovery outcome measures (Neil et al., 2009; Chien and Chan, 2013; Law et al., 2014; Lemos-Giráldez et al., 2015; Williams et al., 2015; Argentzell et al., 2017; Kanehara et al., 2017; Uriarte et al., 2020; Saavedra et al., 2021) (Tables 4, 5), this study is the only one to meet all the minimum standards for PRO measures (Reeve et al., 2013): conceptual and measurement model, translation of the measure, interpretation of scores including “Guidelines for Clinicians, Researchers and Service Users for the uses, administration and scoring of the QPR”; consideration of the participant burden; reliability over 0.70, and content validity, as reported in the cross-cultural adaptation (Goodman-Casanova et al., 2022), criterion and construct validity as shown by the convergent and divergent correlations, and factory analysis.

**Table 4:** Indexes of the QPR-15-SP validation and other QPR versions.

	Goodman-Casanova et al. (2022)	Law et al. (2012)	Williams et al. (2015)	Neil et al. (2009)	Chien and Chan (2013)	Argentzell et al. (2017)	Kanehara et al. (2020)
Measure	15-item Spanish version	15-item version	15-item version	22-item Original version	22-item Chinese version	22-item Swedish version	22-item Japanese version
Sample, women (%)	104 (28.8)	355 (33.7)	487 (32.6)	111 (43.3)	300 (45)	226 (72)	197 (61.9)
Mean (SD)	41.77 (11.143)	50.13 (11.56)	Data-set 1 41.17 (8.6); Data-set 2 37.82 (9.1)	Subscale 1 45.74 (16.1); subscale 2 14 (3.7)	Group 1 48.2 (17.3); Group 2 28.1 (9.5)	73.9 (13.7)	56.8 (12.8)
Internal consistency	*α* = 0.92	*α* = 0.93	*α* = 0.89	Subscale 1 *α* = 0.94; subscale 2 *α* = 0.77	*α* = 0.90	*α* = 0.91	*α* = 0.91
Temporal reliability	ICC = 0.684	*r* = 0.70	ICC = 0.74	Subscale 1 *r* = 0.874; subscale 2 *r* = 0.769	ICC = 0.89	Not reported	ICC = 0.85
Factor analysis	1 factor	1 factor	1 factor	2 factors	3 factors	1 factor	5 factors

**Table 5:** Indexes of the QPR-15-SP validation and other recovery measures in Spain.

	Goodman-Casanova et al. (2022)	Lemos-Giráldez et al. (2015)	Uriarte et al. (2020)	Saavedra et al. (2022)
Measure	QPR-15-SP 15 items	STORI 50 items	REE 24 items	RAS-24 24 items
Sample, women (%)	104 (28.8)	95 (29.5)	312(39.4)	305(44.3)
Internal consistency	*α* = 0.92 *ω* = 0.93	Moratorium *α* = 0.86	*α* = 0.90	*α* = 0.93 *ω* = 0.95
		Awareness *α* = 0.83		
		Preparation *α* = 0.86		
		Rebuilding *α* = 0.83		
		Growth *α* = 0.87		
Temporal reliability	ICC = 0.684	Not reported	Not reported	ICC = 0.89
Factor analysis	1 factor	3 clusters	1 factor	5 factors

Our samples sociodemographic characteristics were similar to those of other international (Neil et al., 2009; Chien and Chan, 2013; Law et al., 2014; Williams et al., 2015; Argentzell et al., 2017; Kanehara et al., 2017), and national validations (Lemos-Giráldez et al., 2015; Uriarte et al., 2020; Saavedra et al., 2021) ensuring the representativeness of the population who uses mental health services.

The reported reason for 6% of the sample to interrupt the assessment when completing the STORI is indicative of the importance of the validation of user-friendly outcome measures, and is in line with other studies which have reported a negative rating for user friendliness of this instrument (Cavelti et al., 2012).

The descriptive statistics show that the studied population felt least recovered in terms of feeling more isolated than part of society, and most recovered in regard to finding time to do the things they enjoyed. These findings are in line with the previous of the cross-cultural adaptation (Goodman-Casanova et al., 2022) and coincide with the marked social and relational character of recovery identified in Spain (Saavedra et al., 2022), and stress the importance of the impact of social support in recovery (Bjørlykhaug et al., 2021).

Internal consistency of the QPR-15-SP was overall higher than the other QPR versions Table 4 and the STORI, but slightly lower than the REE and the RAS-24 (Table 5). The QPR-15-SP scores obtained regarding temporal reliability was somewhat inferior to the rest of the comparable measures (Tables 4, 5).

This is the first validation in Spain to have tested the validity of an instrument with another validated recovery instrument, the STORI, and with a clinical outcomes measure, the CORE-OM, and is also the first study to compare the psychometric properties of the available recovery measures in Spain.

The confirmatory factor analysis showed a reasonable goodness of fit but with less adequacy than the studies that tested the 1-factor solution in the original language (Law et al., 2014; Williams et al., 2015). When it comes to international comparison research adapting an instrument is more efficient than creating a new one. However the heterogeneity and complexity of recovery, and its social and relational character in Spain hinder reaching a more precise cross-cultural factor similarity.

This multicenter study included participants from three different regions in Spain, and from both primary and specialized mental health services contributing to a wider cultural and service-related diversity, and promoting the future planning of intersectoral care by offering a culturally-tailored user-defined recovery outcome measure. Mental health professionals such as mental health nurses, psychologists and psychiatrists are key to recovery action planning and ideally placed to lead the evaluation of mental health services and programs following internationally valid frameworks, such as the CHIME, using validated measures ensuring international comparison research.

It would be interesting for future studies, to compare the acceptability of the available Spanish measures to determine which is more user-friendly, and which best correlates with the CHIME framework. Moreover, quantitative studies of interventions that support recovery will allow a better understanding of changes in recovery over time and exploring sensitivity to change of the measure.

The following limitations of this study should be noted. While the sample was similar to those of other studies, there was a gender-based skew with a higher representativeness of men. This is in line with research which has described a greater representativeness of men with severe mental illness in Spain (Medel-Herrero et al., 2015) and particularly in the context studied (Petkari et al., 2017). There is great variability in this bias amongst the rest of the studies which present sex-disaggregated data, with samples of predominantly men (Neil et al., 2009; Chien and Chan, 2013; Law et al., 2014; Lemos-Giráldez et al., 2015; Williams et al., 2015; Uriarte et al., 2020; Saavedra et al., 2021) versus those which are mostly represented by women (Argentzell et al., 2017; Kanehara et al., 2017). Future research should be gender-sensitive and consider the gender gap in mental health services (Pattyn et al., 2015). Another limitation is that RMSEA was above recommended. When samples are small, the RMSEA often falsely indicates a poor fitting model (Kenny et al., 2014). Moreover, though there is no consensus in defining sample validation sizes of PRO measures (Reeve et al., 2013) and our sample size meets the minimum of 100 participants (Anthoine et al., 2014), the sample size is small and thus, findings should be taken with caution.

The QPR-15-SP has acceptable psychometric properties, providing support for measuring recovery in Spain and allowing international comparison research. The Spanish version of the QPR is a cross-culturally adapted and psychometrically validated quantitative self-administered instrument to measure user-defined recovery-oriented outcomes based on the CHIME framework. This measure seeks to become a useful routine outcome measure of recovery in Spain. The QPR-15-SP may be used in controlled trials of recovery from psychosis providing standardized data, allowing comparison across different populations and testing of the effectiveness of mental health services, programs and interventions over time.

## Data availability statement

The raw data supporting the conclusions of this article will be made available by the authors, without undue reservation.

## Ethics statement

The studies involving human participants were reviewed and approved by Comité de Ética de la Investigación Provincial de Málaga (0385-N-19) and Comité de Ética de la Investigación con medicamentos del Hospital Universitario Príncipe de Asturias (OE 47_2020) Trial registration: NCT03985904 (Goodman-Casanova, et al., 2022). The patients/participants provided their written informed consent to participate in this study.

## Author contributions

JMG-C, DC-L, FM-C, and JG-P made substantial contributions to the conception and design of the work. JMG-C acquisition of data. JMG-C and JG-P analysis and interpretation of data and drafting the work. JMG-C, DC-L, FM-C, and JG-P revising the work critically for important intellectual content and final approval of the version to be published. CA and EG acquisition of data content and final approval of the version to be published with JH-I. All authors contributed to the article and approved the submitted version.

## Funding

This study was carried out under a Río Hortega Contract (CM20-00177), co-financed by the Instituto de Salud Carlos III and by the European Social Fund (ESF) 2014–2020 “The ESF invests in your future.”

## Conflict of interest

The authors declare that the research was conducted in the absence of any commercial or financial relationships that could be construed as a potential conflict of interest.

## Publisher’s note

All claims expressed in this article are solely those of the authors and do not necessarily represent those of their affiliated organizations, or those of the publisher, the editors and the reviewers. Any product that may be evaluated in this article, or claim that may be made by its manufacturer, is not guaranteed or endorsed by the publisher.
