# Supplementary material for: Psychometric validation of the 15-item Questionnaire about the Process of Recovery in Spain (QPR-15-SP)
Source: Front Psychol. 2023 Jul 5;14:1178341. doi: 10.3389/fpsyg.2023.1178341 (PMC10356816; doi:10.3389/fpsyg.2023.1178341)
Supplement: Supplementary file 1 [file Data_Sheet_1.docx]

Supplementary Material

# Supplementary Data

**Versión española del Cuestionario sobre el Proceso de Recuperación (QPR-15-SP): Guía para profesionales clínicos, investigadores y personas usuarias para la utilización, administración y puntuación del QPR-15-SP**

*Desarrollado por: Sandra T. Neil, Liz Pitt, Martina Kilbride, Anthony P. Morrison, Sarah Nothard, Mary Welford y William Sellwood en colaboración con el Comité Directivo de Servicios al Usuario de The Bolton Salford and Trafford*

*Versión en 15 ítems desarrollada por: Heather Law, Sandra T. Neil, Graham Dunn y Anthony P. Morrison*

*Versión traducida, adaptada culturalmente y validada psicométricamente en España por: Jessica Marian Goodman-Casanova, Daniel Cuesta-Lozano, Fermin Mayoral-Cleries, Jose Guzman-Parra.*

**¿Qué es el Cuestionario sobre el Proceso de Recuperación?** Es un cuestionario de 15 afirmaciones desarrollado en colaboración con personas usuarias de servicios de salud mental con diagnóstico de psicosis a partir de sus experiencias en recuperación. La idea del cuestionario es preguntar a personas sobre aspectos de la recuperación que sean significativos para ellas. El cuestionario es fiable y válido y se encuentra fuertemente asociado con el bienestar general psicológico, la calidad de vida y el empoderamiento que son cruciales en el proceso de recuperación en personas con diagnóstico de psicosis.

**¿Cuáles son las aplicaciones del cuestionario?**

- **Práctica clínica:** Dado que el cuestionario pregunta aspectos sobre la recuperación que son importantes para las personas, esta medida puede ayudar a facilitar la comunicación y el compromiso con su proceso de recuperación. El cuestionario puede ser utilizado para mostrar cómo otras personas han progresado para lograr objetivos similares y, este mensaje positivo, puede dar esperanza, lo cual resulta crucial en la recuperación.
- El cuestionario puede ser utilizado tanto como herramienta para establecer objetivos hacia resultados individuales, como para medir los logros alcanzados por dichos objetivos. Por ejemplo, el QPR-15-SP puede ser utilizado para ayudar a personas a expresarse, darles una estructura y ofrecerles un enfoque para trabajar hacia metas individuales, siendo posteriormente utilizadas para hacer un seguimiento del progreso y proporcionar evidencias de ello.
- Actualmente se está evaluando la sensibilidad del cuestionario, para evaluar su uso como medida de efectividad de servicios de salud mental y como una medida de resultado rutinaria de recuperación.
- **Investigación**: Se recomienda que los profesionales investigadores utilicen el cuestionario para ampliar y añadir evidencia basada en el área de la recuperación.

**¿Cómo administrar el cuestionario?** Las personas implicadas en el desarrollo del cuestionario recomiendan que, antes de administrarlo, los clínicos e investigadores que utilicen esta medida se aseguren de que:

- Todas las personas a las que se les proponga completar el cuestionario dispongan de información general (arriba mencionada) y que se les proporcione una explicación de por qué se les pide completar este cuestionario, como por ejemplo “Esperamos que al completar esta evaluación, podamos identificar áreas de su vida en las que van bien las cosas, así como áreas en las que pueda encontrar dificultades".
- Todas las personas deben dar su consentimiento verbal o escrito para completar el cuestionario.
- Siempre que sea posible el cuestionario debe ser cumplimentado con otro profesional o persona con quien poder discutir posibles cuestiones planteadas.
- El cuestionario debe ser utilizado con criterio y responsabilidad clínica y, **no** se les debe pedir que lo completen a aquellas personas que se encuentren en una crisis y/o bajo mucho malestar.
- El cuestionario no debe ser utilizado de forma descuidada, sino como un vehículo para facilitar la discusión de objetivos individuales.

**¿Cómo se puntúa el cuestionario?** El **cuestionario** dispone de 15 afirmaciones, todas ellas evaluadas en una escala sobre 4 puntos (0= Totalmente en desacuerdo, 1= Desacuerdo, 2= Ni de acuerdo ni en desacuerdo, 3= De acuerdo, 4= Totalmente de acuerdo). Puntuaciones elevadas son indicativas de recuperación. Sin embargo, las personas que han desarrollado el cuestionario indican que más allá de la puntuación de recuperación, el QPR-15-SP puede ser utilizado como se ha descrito anteriormente, por ejemplo, como herramienta para potenciar el compromiso con su proceso de recuperación, para establecer objetivos individuales y como medida de resultado de los mismos.

**POR FAVOR, DE LA VUELTA A LA PÁGINA Y CONTINÚE EN EL OTRO LADO**

**Versión española del Cuestionario sobre el Proceso de Recuperación (QPR-15-SP)**

[15/10/2007- Versión 1]

[02.04.2014 Versión 2]

[15.05.2021 - Versión traducida y adaptada culturalmente]

[21.07.2022 - Versión validada psicométricamente]

Este cuestionario fue desarrollado para entender mejor el proceso de recuperación definido como un proceso único que posibilita vivir una vida satisfactoria y plena más allá de la enfermedad y el malestar mental. Las afirmaciones de este cuestionario se desarrollaron a través de entrevistas a personas usuarias de servicios de salud mental sobre su proceso de recuperación. Esperamos que al completar este cuestionario nos ayude a encontrar información importante para usted y para su propia recuperación. No todos los aspectos serán de importancia para usted ya que todos somos diferentes. Este cuestionario no pretende imponerle nada en contra de su voluntad.

Si desea completar el cuestionario, por favor, dedique un momento a considerar y valorar cómo son las cosas para usted en el presente, concretamente en los últimos 7 días, en relación con su salud mental y su recuperación. Por favor, responda a las siguientes afirmaciones marcando con una X la casilla que mejor describa su experiencia.

|  | Totalmente en desacuerdo | Desacuerdo | Ni de acuerdo,  ni en desacuerdo | De acuerdo | Totalmente  de acuerdo |
| --- | --- | --- | --- | --- | --- |
| 1. Me siento mejor conmigo mismo/a |  |  |  |  |  |
| 1. Me siento capaz de aprovechar las oportunidades de mi vida |  |  |  |  |  |
| 1. Soy capaz de desarrollar relaciones positivas con otras personas |  |  |  |  |  |
| 1. Me siento parte de la sociedad y no aislado/a |  |  |  |  |  |
| 1. Soy capaz de defender mis derechos |  |  |  |  |  |
| 1. Siento que mi vida tiene un propósito |  |  |  |  |  |
| 1. Mis experiencias me han cambiado para mejor |  |  |  |  |  |
| 1. He sido capaz de aceptar las cosas que me han ocurrido en el pasado y seguir adelante con mi vida |  |  |  |  |  |
| 1. Estoy fuertemente motivado/a para mejorar |  |  |  |  |  |
| 1. Puedo reconocer las cosas positivas que he hecho |  |  |  |  |  |
| 1. Soy capaz de entenderme mejor |  |  |  |  |  |
| 1. Puedo responsabilizarme de mi vida |  |  |  |  |  |
| 1. Puedo implicarme activamente en mi vida |  |  |  |  |  |
| 1. Puedo tomar el control de aspectos de mi vida |  |  |  |  |  |
| 1. Puedo encontrar tiempo para hacer las cosas que disfruto |  |  |  |  |  |

**Gracias por completar este cuestionario**

**©Neil et al, 2007 (No se puede reproducir sin permiso de sus autores)**

**©V2 Neil, et al 2014.**

**Goodman-Casanova et al, 2021 (Traducción y adaptación cultural en España)**

**Goodman-Casanova et al, 2022 (Validación psicométrica en España)**

**Adaptado con permiso de Sandra Neil. Original English versión available at:**

https://www.rcpsych.ac.uk/docs/default-source/improving-care/ccqi/quality-networks/early-intervention-in-psychosis-teams-(eipn)/eipn-questionnaire-about-the-process-of-recovery-15-item.pdf?sfvrsn=a754873b_2
